# Rapid and simple detection of *Candida albicans* using closed dumbbell-mediated isothermal amplification

**DOI:** 10.3389/fcimb.2025.1484089

**Published:** 2025-02-03

**Authors:** Yanli Zhang, Xuhan Chen, Yeling Zhong, Fei Guo, Guifang Ouyang, Rui Mao

**Affiliations:** ^1^ Department of Hematology, The First Affiliated Hospital of Ningbo University, Ningbo, Zhejiang, China; ^2^ Ningbo Institute of Life and Health Industry, University of Chinese Academy of Sciences, Ningbo, China; ^3^ Department of General Surgery (Hepatic, Anal-canal, Gastrointestinal), Ningbo Zhenhai People’s Hospital, Ningbo, Zhejiang, China; ^4^ Department of Laboratory Medicine, The First Affiliated Hospital of Ningbo University, Ningbo, Zhejiang, China

**Keywords:** *Candida albicans*, point-of-care diagnostic, closed dumbbell mediated isothermal amplification, real-time fluorescence CDA, visual CDA, sensitivity, specificity

## Abstract

**Introduction:**

*Candida albicans*, a human fungal pathogen, multiplies to invade body cells and causes fungal diseases in the condition of insufficient body's immune function. Early detection of *C. albicans* is required to guide appropriate prevention and treatment.

**Methods:**

The purpose of this study was to establish a *C. albicans* assay based on newly developed closed dumbbell-mediated isothermal amplification (CDA) to achieve rapid and simple point of care diagnostic. The CDA technique was carried out by specific primers targeting at the conserved *C. albicans* ITS2 gene. All primers were selected and evaluated by real-time fluorescence monitoring and endpoint visual judgement indicated by hydroxy naphthol blue (HNB). Optimal primers and accelerate primers (out primers and loop primers) were designed and selected after confirmation of the fundamental CDA primers to achieve more efficient CDA reaction for *C. albicans* detection (CA-OL-CDA).

**Results:**

After establishment of the assay, 9 *non-Candida albicans* strains, including 3 *Candida* species were tested to negative by adopting the established CA-OL-CDA assay, indicated high specificity. The limit of detection of *Candida albicans* DNA by CA-OL-CDA assay was 6.2×10^-6^ ng/μL of DNA (10 copies/μL), 10-fold more sensitive than real-time quantitative PCR (qPCR).

**Discussion:**

The CA-OL-CDA assay exhibited advantages of high specificity, sensitivity, simpler and more efficient operation. In addition, the CA-OL-CDA method holds potential in on-site detection for *C. albicans* using color shift by adopting the reaction mixture based on HNB.

## 1 Introduction

*Candida albicans* is the most prevalent human fungal pathogen and a commensal in a large portion of the population, colonizing the oral, gastrointestinal, and reproductive tracts asymptomatically (Wall et al., 2019; Ho et al., 2021; Wakade et al., 2021). Usually, *C. albicans* could invade body cells and cause disease under abnormal immune function conditions (Kadosh, 2016; Bertolini et al., 2019; Lopes and Lionakis, 2022). As reported in the United States, approximately 70% of women may suffer from *C. albicans* vaginal infection during their lifetime (Nyirjesy et al., 2022). In addition, burn wounds may contribute to candidiasis via high colonization and infection by *C. albicans* (Fidel et al., 1999; Wisplinghoff et al., 2004; von Müller et al., 2020; Brandenburg et al., 2021). Moreover, various predispositions lead to the occurrence of candidiasis, including AIDS, steroid therapy, organ and tissue transplantation, cancer therapy, and broad-spectrum antibiotics (Hsu et al., 2021; Kumwenda et al., 2022; Liu et al., 2022). Therefore, it is significant to detect *C. albicans* accurately for the prevention, control, and7nbsp;efficient treatment of the disease caused by this conditioned pathogen.

Currently, polymerase chain reaction (PCR) has been the most commonly used method for the diagnosis of *C. albicans* (Wahyuningsih et al., 2000). However, PCR detection proved relatively complex to some field conditions and special environments because of expensive thermocycles, well-trained operators, and gel electrophoresis instruments (Farrar and Wittwer, 2015). In recent years, various isothermal amplification techniques that offer alternative approaches to the well-established PCR-based methods for *C. albicans* detection have been developed, such as loop-mediated isothermal amplification (LAMP), polymerase spiral reaction (PSR), and recombinase polymerase amplification (RPA) (Jiang et al., 2016; Noguchi et al., 2017; Wang et al., 2021). Among the isothermal amplification methods discussed above, owing to the advantages of high specificity, simplicity, high sensitivity, and rapid accumulation of targeted DNA, LAMP and the modified LAMP method have been adopted for commercial assays (Noguchi et al., 2017; Lin et al., 2021; Ou et al., 2021; Tu et al., 2024). However, the drawbacks of LAMP lie in its sophisticated, cumbersome, and complex primer design, which makes further expansion and application inconvenient (Gonçalves Dda et al., 2014; Mao et al., 2018; Gomez-Gutierrez and Goodwin, 2022). Thus, a novel isothermal nucleic acid amplification method that has a simpler primer design and is more cost-effective for the rapid and sensitive detection of *C. albicans* is needed.

Closed dumbbell-mediated isothermal amplification (CDA) is based on self-primer-mediated single nucleic acid annealing and extending, which was characterized by efficiency, high sensitivity, and high specificity (Gui et al., 2022; Mao et al., 2022; Zhang et al., 2024). In this study, the conserved internal transcribed spacer 2 (ITS2), a region between 5.8S and 28S ribosomal DNA of *C. albicans*, was selected as the target for specific identification and amplification. The specificity and sensitivity of the developed CDA were compared with those of real-time quantitative PCR (qPCR) under similar conditions. In addition, we also successfully assessed the practical application of the CDA assay in real-time fluorescence and on-site visible detection of *C. albicans* with 100% sensitivity and specificity. All results showed that the established CDA assay for *C. albicans* identification had the potential to achieve efficient, rapid, accurate, and visible on-site detection.

## 2 Materials and methods

### 2.1 Ethics statement

Ethical approval, including the waiver of informed consent of the clinical strains and samples collected in the laboratory of the First Affiliated Hospital of Ningbo University, was granted by the Ethics Committee of Clinical Investigation in the First Affiliated Hospital of Ningbo University, under approval number 2024-096A-02. The research conformed to the principles of the Helsinki Declaration. The study involved no more than minimal risk to subjects, and no personal information was obtained.

### 2.2 Isolation of DNA of strains

Thirty clinical isolates of *C. albicans* were collected from July 2023 to August 2024 by the research team from the laboratory of the First Affiliated Hospital of Ningbo University, and various non-*C. albicans* clinical isolates and strains were listed. Standard strains of *C. albicans* (CICC 1965), *Candida glabrata* (BNCC 337348), *Candida parapsilosis* (BNCC 337317), *Candida tropicalis* (BNCC 186815), *Klebsiella pneumoniae* (BNCC 361251), *Escherichia coli* (CGMCC, 1.12883), *Staphylococcus aureus* (CGMCC: 1.6750), *Salmonella typhimurium* (CGMCC: 1.1190), *Listeria monocytogenes* (CGMCC: 1.9144), *Vibrio parahaemolyticus* (CGMCC: 1.1997), and *Shigella sonnei* (CVCC 3926) were obtained from the China Center of Industrial Culture Collection (CICC), the BeNa culture Collection (BNCC), the China Gene Microbiological Culture Collection Center (CGMCC), and the China Veterinary Culture Collection Center (CVCC). *Candida* strains were cultured in yeast peptone dextrose (YPD) medium (Li et al., 2021), and *Escherichia coli* strains were cultured in Luria-Bertani (LB) medium (Jira et al., 2018). DNA was extracted from bacteria liquid at 180 rpm for 24 h using DNA purification kits (TIANGEN, Shanghai) and stored at −20°C.

### 2.3 CA-CDA primer design

**Figure 1:**
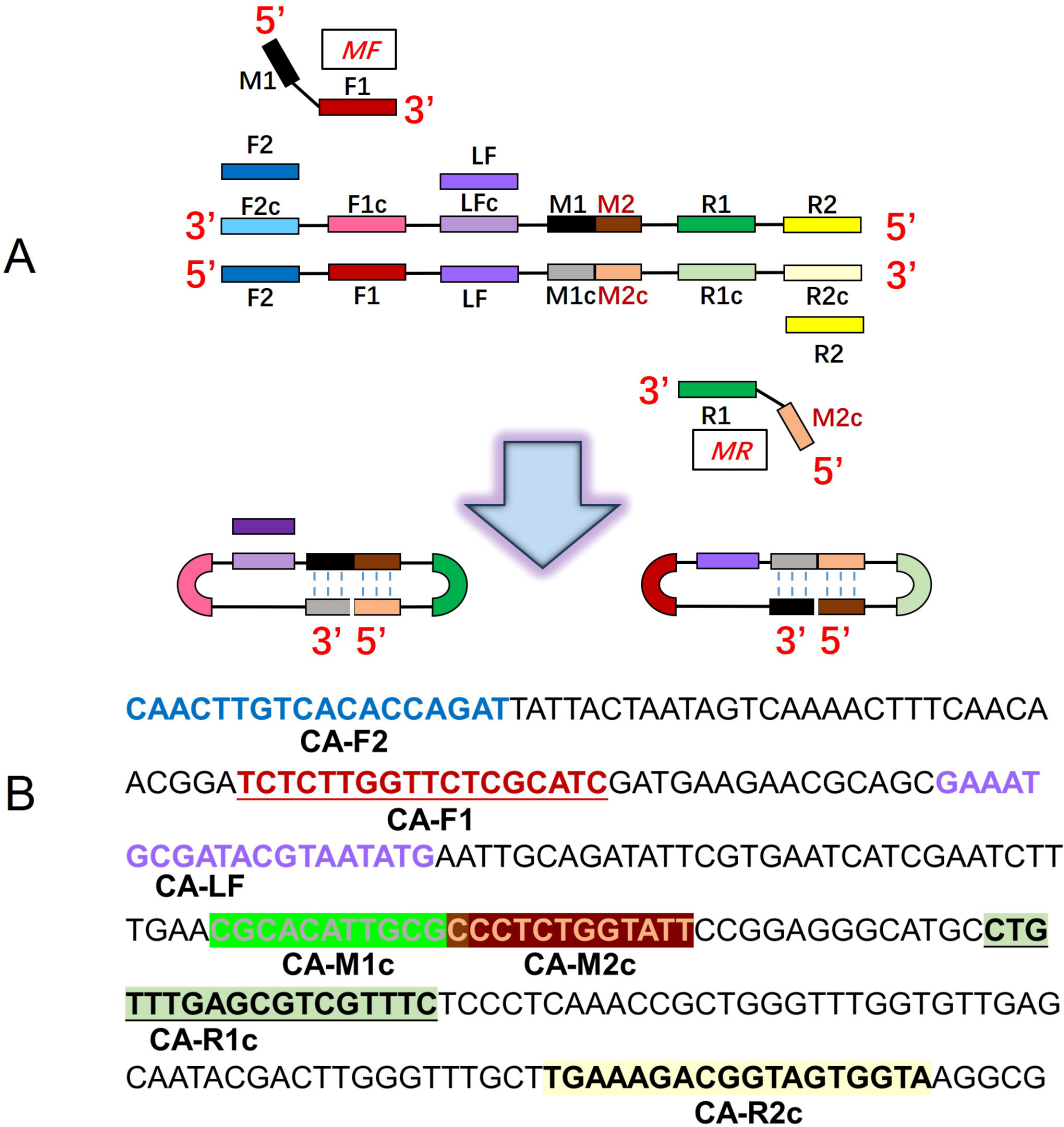
Principle of the closed dumbbell-mediated isothermal amplification (CDA) method. **(A)** Overall scheme for primers used in the CDA method. **(B)** Partial sequences of the *C*. *albicans* ITS2 gene were used to design primers for the CDA method.

Four pairs of primers of *C. albicans* CDA (CA-CDA) were designed targeting the specific conserved sequences of the ITS2 region (Accession No. MT193530.1) after BLAST on the NCBI GenBank database using the DNAMAN Version 8.0 software (**Supplementary Table 1**). The situation of CA-CDA primers on ITS2 sequences is shown in **Figure 1A**. The F1, R1, and M sequences are abbreviations for “forward 1”, “reverse 1”, and “middle”, respectively. Sites with a lowercase “c” stand for “complementary”. M is the middle of F1c and R1, separated into M1 and M2. Under this system, the forward primer (MF) contains sequences complementary to M1 (M1c) and sequences complementary to F1c (F1). The backward primer (MR) contains sequences (M2) complementary to M2c and R1. To achieve further optimization, external primers (F2 and R2) and loop primers (LF) are designed to accelerate the CDA reaction. The location of all CA-CDA primers used in this study is marked with different colors, as shown in **Figure 1B**. The primer sequences are listed in **Table 1** and synthesized by BGI Bioengineering Technical Services Co., Ltd. (Shenzhen, China).

**Table 1:** *Candida albicans* CDA primers and PCR primers targeting the ITS2 gene.

Target	Method	Primer	Sequence (5′→3′)
*Candida albicans* ITS2 gene	CDA	CA-MF	CGCAATGTGCGTTCTCTTGGTTCTCGCATC
		CA-MR	CCCTCTGGTATTGAAACGACGCTCAAACAG
		CA-F2	CAACTTGTCACACCAGAT
		CA-R2	TGAAAGACGGTAGTGGTA
		CA-LF	GAAATGCGATACGTAATATG
	PCR	CA-PF	CGGCTCTTGTCTATGTTCC
		CA-PR	TCATCGCACGGGATTCTCA

### 2.4 CDA assays

CDA assays were carried out as reported by our group (Mao et al., 2022). In short, each CDA reaction was conducted in 25 μL of reaction mixture, including 12.5 μL of 2× isothermal amplification buffer [including 40 mM Tris-HCl, 20 mM KCl, 20 mM (NH_4_)_2_SO_4_, 16 mM MgSO_4_, and 0.2% Triton X-100], 1 μL of *Bst* 2.0 WarmStart DNA polymerase (New England Biolabs), 1 μL of MF and MR, 1 μL each of Eva green and HNB (Biotium, Hayward, CA, USA), and an appropriate amount of nucleic acid template and nuclease-free water. Finally, the reaction mixture was covered with paraffin oil (Shanghai Bioengineering Co., Ltd.). After the addition of the accelerate primers (outer primers and loop primers) with the system of F2 (0.2 μM), R2 (0.2 μM), and LF (0.2 μM), the assay was named CA-OL-CDA. The negative control (NC) contained nuclease-free water. The reaction was incubated at 60–65°C for 60 min and 85°C for 10 min in the CFX96 Real-Time PCR detection system (Bio-Rad, California, USA) and Q3 Real-Time PCR instrument (Applied Biosystems, California, USA). Using the direct visual judgment approach, the color change from purple to sky blue for positive samples was visible by the naked eye under natural light, while the negative reaction remained purple.

### 2.5 CA-OL-CDA sensitivity and specificity analysis

To confirm the specificity of the CA-OL-CDA assay in positive samples of *C. albicans*, we performed the method using three other *Candida* species, i.e., *C. glabrata*, *C. parapsilosis*, and *C. tropicalis*, and six non-*Candida* bacteria, namely, *Staphylococcus aureus*, *Escherichia coli, Shigella sonnei*, *Listeria monocytogenes*, *Vibrio parahaemolyticus*, and *Klebsiella pneumoniae*. All of them were used for the specificity test after the determination of the optimal temperature of 60°C, and sterilized enzyme-free water was deemed as the negative control. The specificity of CA-OL-CDA results was obtained by analyzing the amplification curve and melting curve.

To determine the sensitivity of the CA-OL-CDA method, a 10-fold concentration dilution was prepared from the original concentration of *C. albicans*, and the DNA concentrations were 6.2×10^−1^, 6.2×10^−2^, 6.2×10^−3^, 6.2×10^−4^, 6.2×10^−5^, 6.2×10^−6^, and 6.2×10^−7^ ng/μL, respectively. The sensitivity of the CA-OL-CDA assay was repeated four times to determine the minimum value of template DNA by real-time fluorescence amplification monitoring and visual endpoint judgment. Visual judgment is usually conducted with a white paper under the tubes to avoid mistakes. Furthermore, the amplified products were analyzed by agarose gel electrophoresis.

### 2.6 Real-time quantitative PCR reaction

The real-time qPCR assay was performed using primers F1 (5′-CGGCTCTTGTCTATGTTCC-3′) and R1 (5′-TCATCGCACGGGATTCTCA-3′) (**Figure 1**, **Table 2**). The samples of qPCR were in accordance with the CA-OL-CDA assay and as a comparison and confirmation for the CA-OL-CDA assay results. The total 50-μL volume in qPCR reaction contains 25 μL of 2× Taq PCR Mix (Sangon, Shanghai, China) and 10 mM each of primer 1 × Eva green and the DNA sample. Real-time fluorescence monitoring was carried out using the CFX96 Real-Time PCR detection system (Bio-Rad, California, USA) and a Q3 Real-Time PCR instrument (Applied Biosystems, California, USA). PCR reaction procedures were set as follows: denaturation at 95°C for 2.5 min, annealing at 55°C for 30 s, and extension at 72°C for 20 s for 35 cycles. Finally, the PCR products were subjected to melting curve analysis in the same equipment.

**Table 2:** Determination of sensitivity and specificity of the CA-OL-CDA assay for *Candida albicans*.

Species	Sample numbers		Sensitivity	Specificity	Accuracy
	CA-OL-CDA	Defined	95% CI *	95% CI *	95% CI *
*Candida albicans*	144	144	1.0 (97.5–100.0)	1.0 (98.7–100.0)	1.0 (99.1–100.0)
*Candida glabrata*	0	32			
*Candida tropicalis*	0	32			
*Candida parapsilosis*	0	32			
*Staphylococcus aureus*	0	32			
*Escherichia coli*	0	32			
*Shigella sonnei*	0	32			
*Listeria monocytogenes*	0	32			
*Vibrio Parahaemolyticus*	0	32			
*Klebsiella pneumoniae*	0	32			
Total	144	432			

*CI, confidence interval. Statistical analysis was carried out by the online program “Diagnostic test evaluation calculator” (https://www.medcalc.org/calc/diagnostic_test.php).

## 3 Results

### 3.1 Design and screening of CDA primers for *C. albicans*

**Figure 2:**
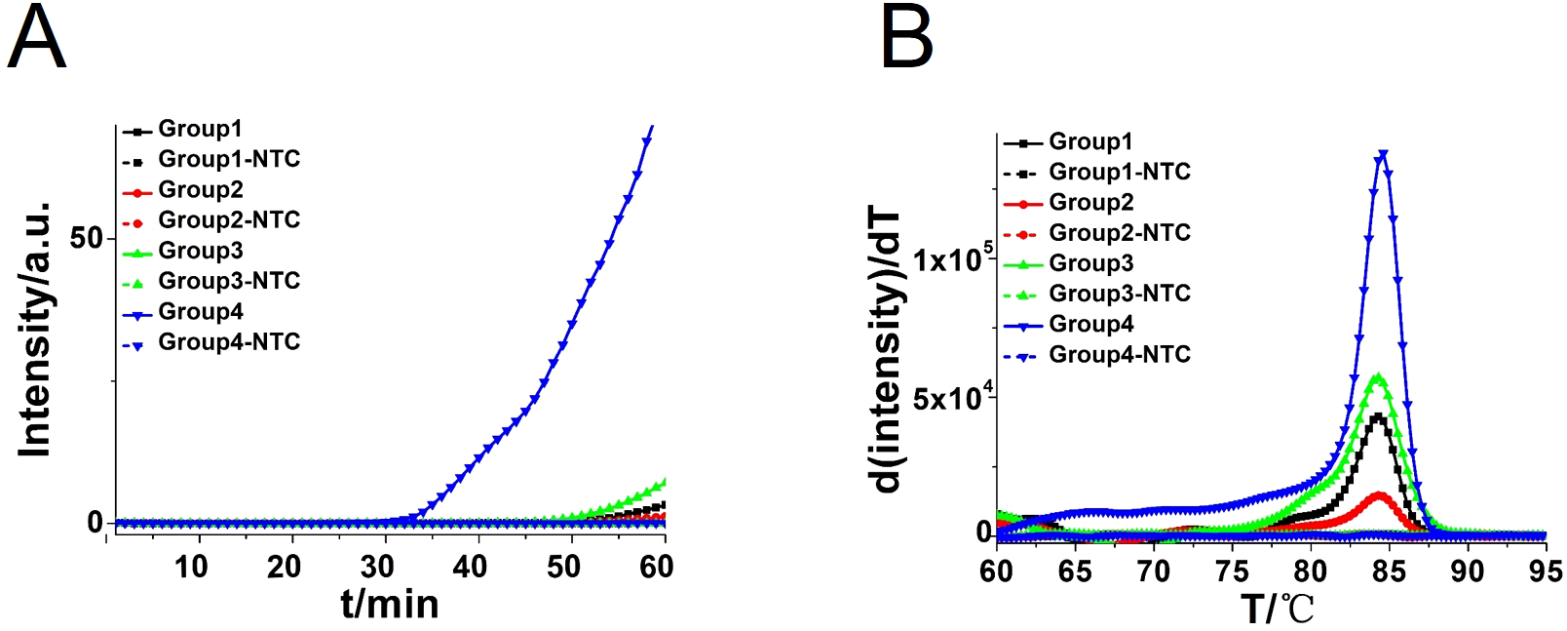
CDA amplification at 60°C for 60 min. **(A)** Real-time fluorescence of different CDA primer groups targeting the ITS2 gene; the primer groups are listed in **Supplementary Table S1**. **(B)** Melting curve analysis of CA-CDA products by real-time PCR. NTC, No template control.

Four pairs of CA-CDA primers targeting at the conserved ITS2 gene of *C. albicans* were amplified to obtain the optimal primer set monitored by the amplification curve and melting curve. The cycle threshold of the CA-CDA reaction was negatively correlated to the amplification efficiency. As shown in **Figure 2**, the amplification curve of the CA-CDA method showed that the fourth group of primers had the best efficiency in amplification. The threshold detection time of 6.2×10^−2^ ng/μL of the *C. albicans* gene was 23 min, the melting temperature of the amplification product was 83.5°C, and the negative control showed no evidence of non-specific amplification.

### 3.2 Optimization of the CA-OL-CDA primer set for *C. albicans*

**Figure 3:**
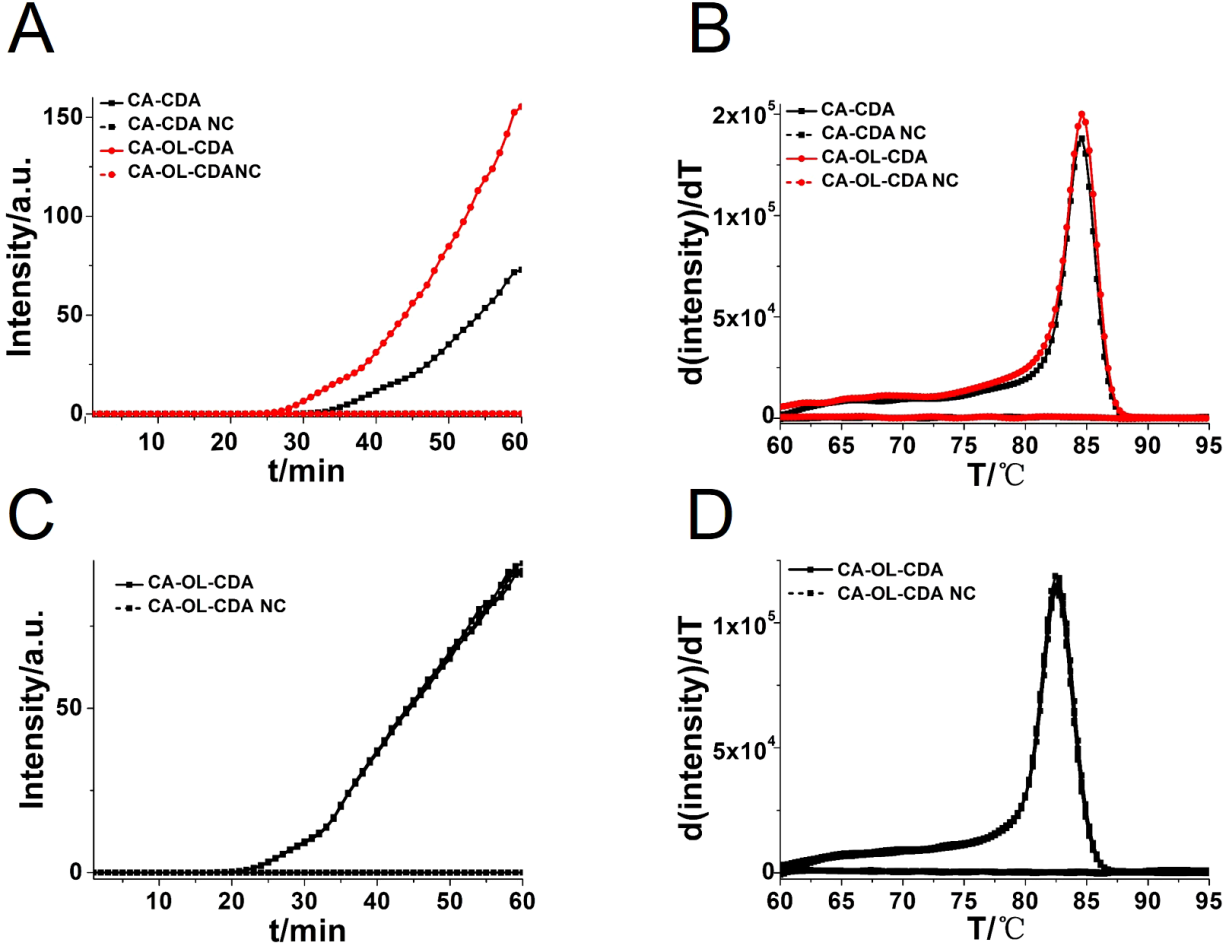
Real-time fluorescent amplification curves of *C*. *albicans* DNA by CA-CDA and CA-OL-CDA. **(A)** Amplification curve of the CA-CDA and CA-OL-CDA assay. **(B)** Melting curve analysis of CA-CDA and CA-OL-CDA results. **(C)** Amplification curve of eight repeated experiments of CA-OL-CDA. **(D)** Melting curve of eight repeated experiments of CA-OL-CDA. PC, positive control. NC, negative control.

To achieve a more efficient CDA amplification process, outer primers and loop primers were designed and verified using the previous optimal primer named CA-OL-CDA. As shown in **Figure 3A**, the reaction efficiency of the CDA method was improved by the addition of accelerate primers, and the threshold time of the CA-OL-CDA method for the detection of *C. albicans* (6.2×10^−2^ ng/μL) was shortened by 6 min. Furthermore, both eight positive and negative replicates were used to evaluate the repeatability of the developed assay. The results of the real-time amplification curve (**Figure 3C**) and the melting curve (**Figure 3D**) presented good repeatability. The melting curves in **Figures 2B**, **3D** presented similar results, indicating a positive effect after the addition of accelerate primers and the absence of non-specific amplification.

**Figure 4:**
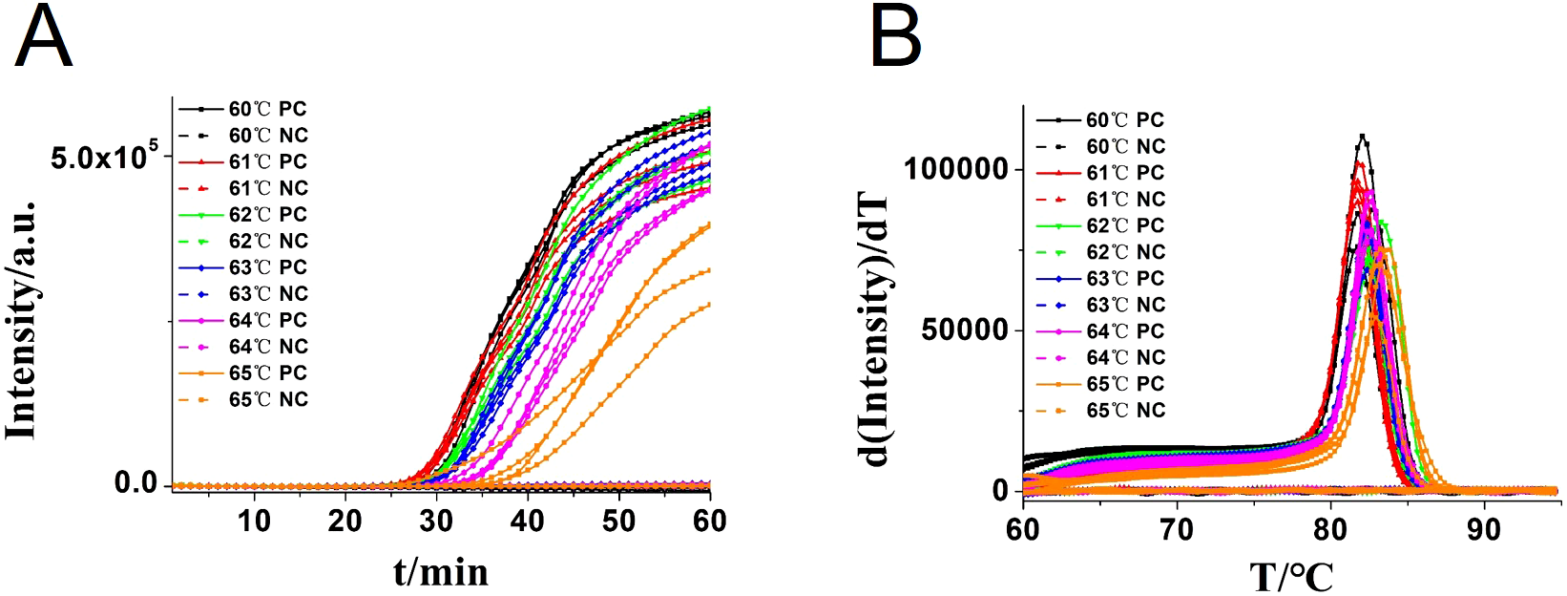
Real-time fluorescent amplification of CA-OL-CDA at different temperatures. **(A)** Amplification curve. **(B)** Melting curve analysis of products. Each set has four positive reactions and four negative controls (NC).

In addition, the reaction temperature for the CA-OL-CDA assay of *C. albicans* was compared and confirmed that the optimal temperature after incubation ranged from 60 to 65°C at 1°C increments. The optimal reaction temperature for the developed CA-OL-CDA method was set at 60°C to ensure positive detection of DNA at low concentrations (**Figure 4**). The results of the real-time amplification curve and the melting curve further supported the good repeatability of the CA-OL-CDA assay.

### 3.3 Assessment of sensitivity of CA-OL-CDA and real-time fluorescence PCR for *C. albicans*

**Figure 5:**
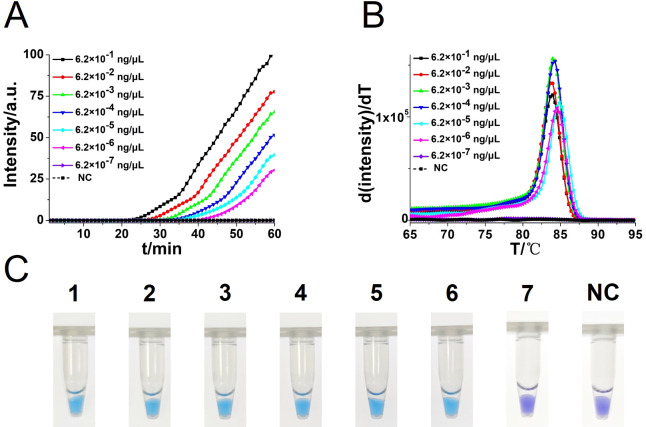
Sensitivity analysis of the CA-OL-CDA method was performed by real-time fluorescent monitoring and visual endpoint judgment. **(A)** CA-OL-CDA amplification was monitored by real-time PCR per 1 min with different concentrations of DNA. The reaction was carried out at 60°C for 60 min. **(B)** Melting curve analysis of *C*. *albicans* OL-CDA products after amplification of different concentrations of extracted DNA. **(C)** Sensitivity analysis of the CA-OL-CDA method for visual detection of *C*. *albicans* DNA. DNA concentrations were as follows: 6.2×10^−1^, 6.2×10^−2^, 6.2×10^−3^, 6.2×10^−4^, 6.2×10^−5^, 6.2×10^−6^, and 6.2×10^−7^ ng/μL and negative control (nuclease-free water).

**Figure 6:**
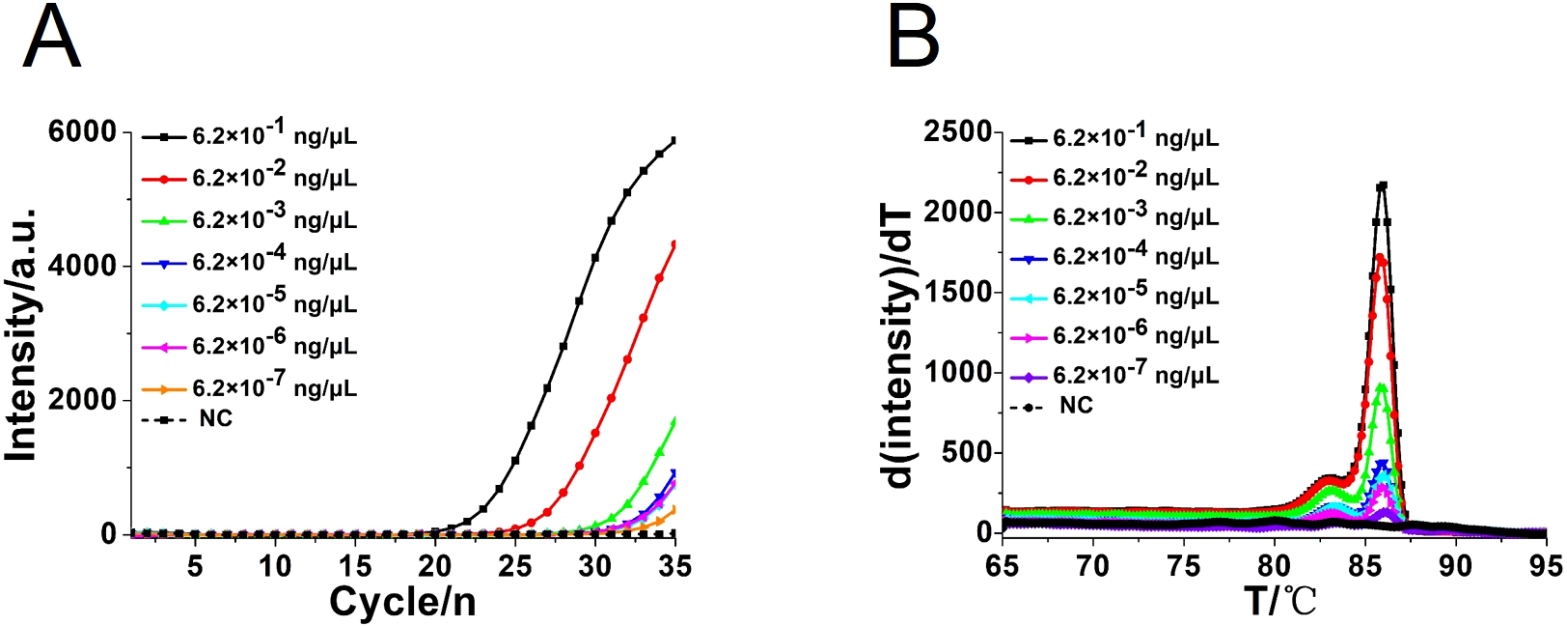
Real-time PCR assay for *C*. *albicans* DNA detection. **(A)** Real-time amplification curve. **(B)** Melting curve analysis of *C*. *albicans* by real-time PCR assay. NC, negative control reaction without the template.

To compare the detection sensitivity of the developed CA-OL-CDA assay and real-time qPCR assay, the same concentrations of 10-fold serial dilutions of DNA of *C. albicans* ranging from 6.2×10^−1^ to 6.2×10^−7^ ng/μL were tested. As shown by the real-time fluorescence plot and the color change of the reaction mixture, the minimum limit of detection of *C. albicans* by CA-OL-CDA was 6.2 fg/μL DNA (**Figures 5A, C**; **Supplementary Figure 1**), which is 10 times more sensitive than qPCR for the detection of *C. albicans* (**Figure 6A**). The melting curve analysis of CA-OL-CDA products showed that *T*_m_ was 83.50°C, and the *T*_m_ value shift in lower concentrations may be caused by limited products in a short time. The results showed that the CA-OL-CDA assay is highly sensitive for the detection of the targeted *C. albicans* DNA sequence. In addition, melting curve results by CA-OL-CDA and qPCR showed no difference in *T*_m_ values (**Figures 5B**, **6B**).

### 3.4 Specificity of the CA-OL-CDA assay

**Figure 7:**
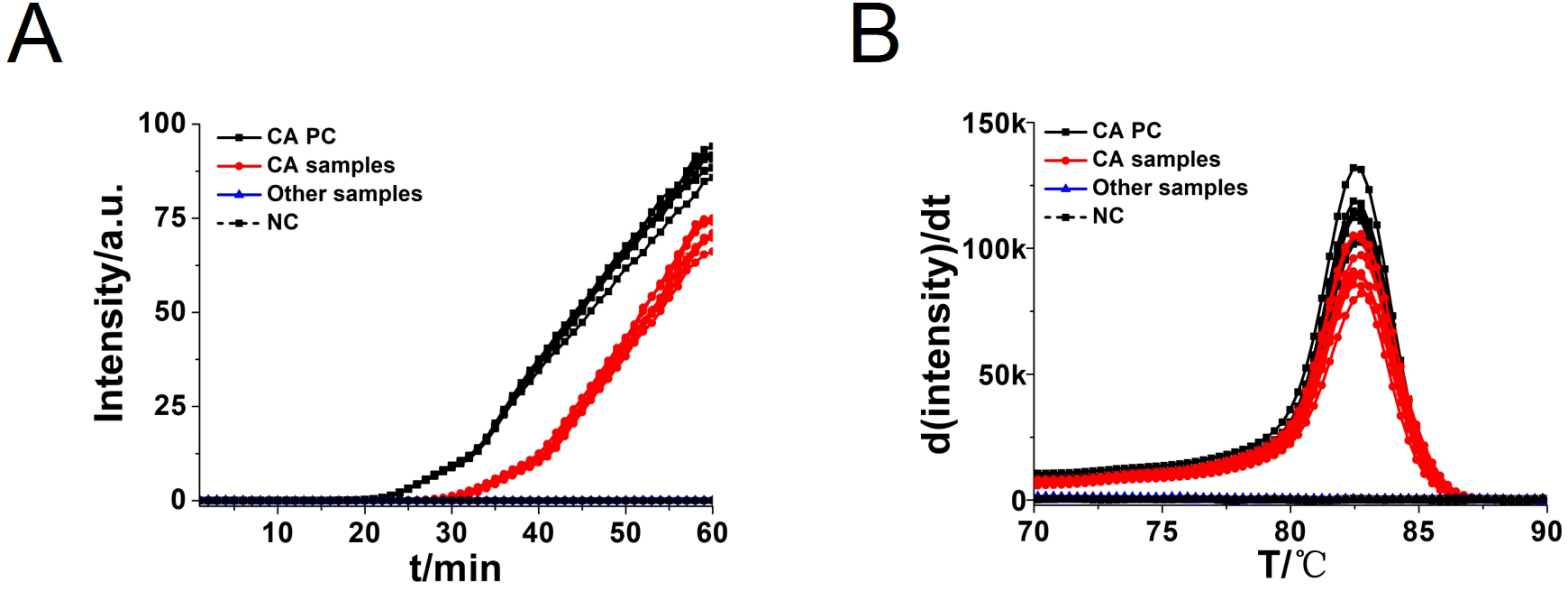
Real-time fluorescent amplification *C. albicans* OL-CDA assay. Real-time CA-OL-CDA reactions were carried out at 60°C for 60 min. **(A)** Real-time CA-OL-CDA for the *Candida albicans* positive control (CA PC), different *Candida albicans* samples, other samples (*Candida glabrata*, *Candida parapsilosis*, *Candida tropicalis*, *Staphylococcus aureus*, *Escherichia coli*, *Shigella sonnei*, *Listeria monocytogenes*, *Vibrio parahaemolyticus*, and *Klebsiella pneumoniae*), and no template control (NC). **(B)** Melting curve analysis of CA-OL-CDA products. PC, positive control. NC, negative control.

**Figure 8:**
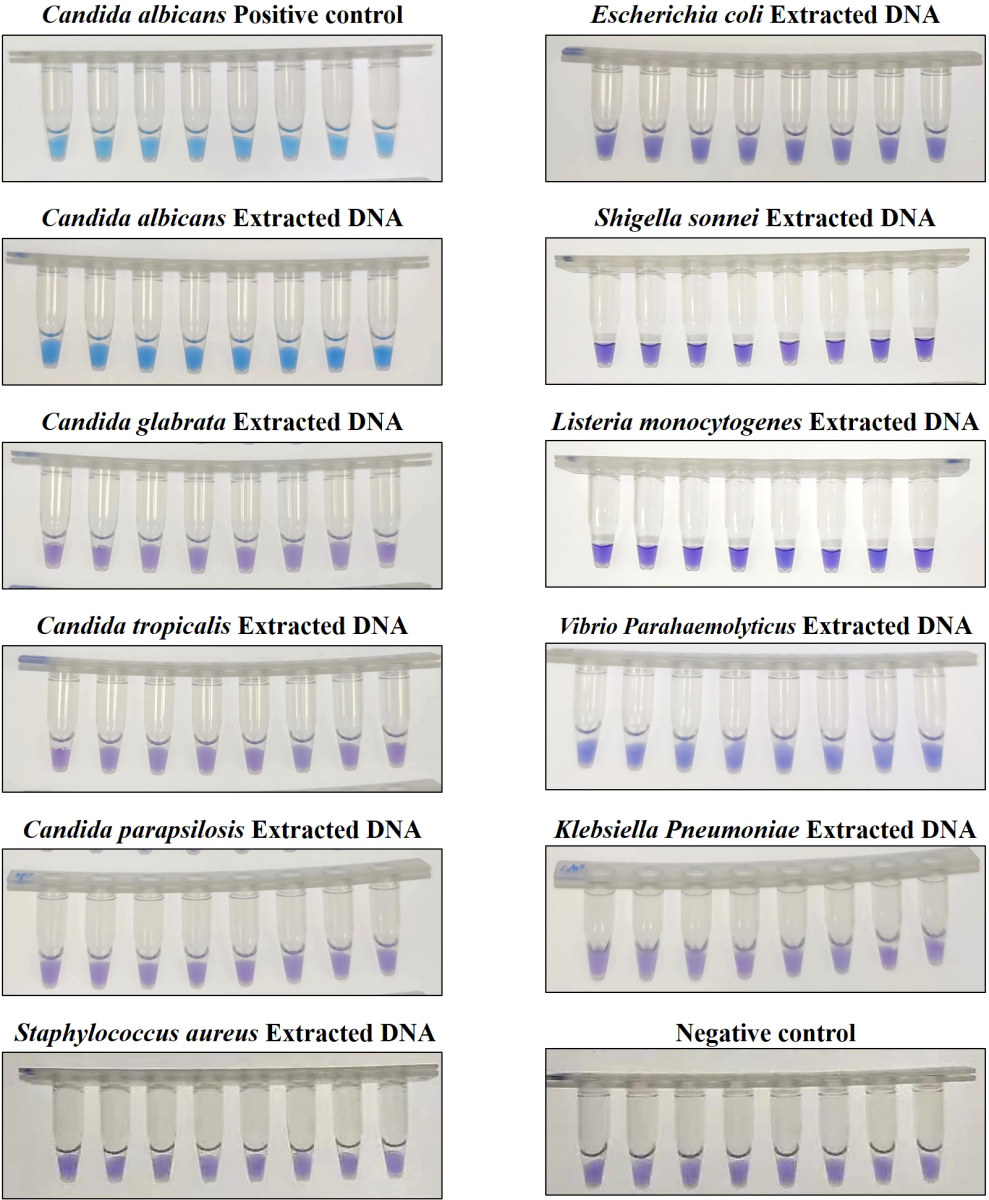
Endpoint colorimetric CA-OL-CDA method using HNB. *Candida albicans* positive controls (6.2×10^−2^ ng/μL of templates). Extracted DNA samples from *Candida albicans*, *Candida glabrata, Candida parapsilosis, Candida tropicalis, Staphylococcus aureus, Escherichia coli, Shigella sonnei, Listeria monocytogenes, Vibrio parahaemolyticus, and Klebsiella pneumoniae*.

To assess the specificity of the CA-OL-CDA method, *C. albicans* (CICC 1965) were set as a positive control. In addition, *C. glabrata*, *C. parapsilosis*, and *C. tropicalis* and six non-*Candida* strains were tested. The results showed that the obtained CA-OL-CDA primers specifically identified and amplified the targeted DNA sequences (**Figure 7A**). Melting curve analysis showed that *T*_m_ was 83.50°C (**Figure 7B**), and no difference was observed between the positive control and different DNA samples, which was consistent with previous results shown in **Figures 3B, D**, **5B**, exhibiting good reproducibility. As shown in **Figures 7**, **8**, both real-time fluorescence plot and visual detection accurately identified *C. albicans*. All other strains tested negative (**Table 2**).

## 4 Discussion

*C. albicans* is a frequently opportunistic pathogen of humans, with an abnormal growth under an imbalanced environment. With the widespread use of hormones, broad-spectrum antibiotics, and immunosuppressive agents, the infection rate of *C. albicans* has shown an increasing trend (He et al., 2021). The mortality rate due to *C. albicans* infections has been reported to be 40% (Gunsalus et al., 2016). Traditional molecular diagnosis methods of *C. albicans* are somewhat limited owing to complex operations and the lack of accuracy (Dadar et al., 2018). Thus, rapid and accurate detection of *C. albicans* is essential for the prompt treatment of the disease.

Commercialized CHROMagar™ *Candida* and germ tube production are two conventional methods for the identification of *C. albicans*. Both are not accurate, and even the germ tube production method occasionally provides false-negative results (Kim et al., 2002; Wohlmeister et al., 2017). Gradually, nucleic acid-based assays with the advantages of obtaining fast results and being accurate have been applied to detect *C. albicans* with high sensitivity and specificity (Fidler et al., 2018; Fallahi et al., 2020). In contrast, PCR has drawbacks such as high cost, inconvenience with the use of instruments, and the possibility of obtaining false-negative results via the PCR assay (sensitivity: 94.1%) (Jiang et al., 2016). In addition, the well-established LAMP has been applied to detect a variety of pathogens, such as *Escherichia coli*, *Klebsiella pneumoniae*, *Pseudomonas aeruginosa*, and *C. albicans*. However, we found that LAMP, which has a complicated primer design and provides false-positive results, was not convenient to carry out (Fallahi et al., 2020; Griffioen et al., 2020; Rivoarilala et al., 2021).

In this study, we successfully developed a CA-OL-CDA assay with high sensitivity (100%), high specificity (100%), and high accuracy that was simpler to use and more efficient for the detection of *C. albicans* compared with PCR-based approaches. We amplified 6.2×10^−2^ ng/μL of the *C. albicans* DNA within 60 min at isothermal 60°C with good reproducibility. Sensitivity experiments have demonstrated that the lower limit of detection of CA-OL-CDA is 6.2 fg/μL of extracted *C. albicans* DNA, and the detection sensitivity is 10 times lower than that of qPCR. The commercial *C. albicans* LAMP kits aimed at oral exfoliative cytology samples is capable of detecting 1 pg per reaction (Noguchi et al., 2017). In addition, the time required for CA-OL-CDA is shorter than that of the qPCR method. Comparison of the results obtained by the CA-OL-CDA technique and the results of real-time PCR methods for *C. albicans* showed that the CA-OL-CDA technique could detect *C. albicans* with relatively equal sensitivity and specificity to real-time PCR tests without the disadvantages of the real-time PCR method. Specificity tests revealed that the CA-OL-CDA method did not identify closely related *C. glabrata*, *C. parapsilosis*, and *C. tropicalis*. No cross-reactivity was also observed for the six distantly related *S. aureus, E. coli, S. sonnei, L. monocytogenes, V. parahaemolyticus*, and *K. pneumoniae*. The detection result can be judged by observing the color difference of the reaction mixture, which has great potential for on-site detection. Moreover, the amplification curves, melting curve, and endpoint visual judgments of 144 batches of positive tests and 288 batches of negative tests were consistent.

## 5 Conclusions

A sensitive and specific CDA method for the visual detection of *C. albicans* was established, which can be applied for on-site detection in areas with relatively developing medical conditions. The CA-OL-CDA method may help to improve the detection efficiency and the prevention and medication of diseases caused by *C. albicans*.

## Data Availability

The original contributions presented in the study are included in the article/**Supplementary Material**. Further inquiries can be directed to the corresponding author/s.
