# Supplementary material for: Rapid and simple detection of Candida albicans using closed dumbbell-mediated isothermal amplification
Source: Front Cell Infect Microbiol. 2025 Feb 3;15:1484089. doi: 10.3389/fcimb.2025.1484089 (PMC11830661; doi:10.3389/fcimb.2025.1484089)
Supplement: Supplementary file 1 [file Table1.docx]

Supplementary Material

Rapid and simple detection of *Candida albicans* using closed dumbbell mediated isothermal amplification

**Yanli Zhang ^1^**^,^ **^+^, Xuhan Chen ^2^**^,^ **^+^, Yeling Zhong ^3^,** **Fei Guo ^4^, Guifang Ouyang ^1, *^ and Rui Mao ^2, *^**

^1^ Department of Hematology, The First Affiliated Hospital of Ningbo University, Ningbo 315010, Zhejiang, China

^2^ Ningbo Institute of Life and Health Industry, University of Chinese Academy of Sciences, Ningbo 315010, China.

^3^ Department of General Surgery (Hepatic, Anal-canal, Gastrointestinal), Ningbo Zhenhai People’s Hospital, Ningbo 315020, Zhejiang, China

^4^ Department of Laboratory Medicine, The First Affiliated Hospital of Ningbo University, Ningbo 315010, Zhejiang, China.

*** Correspondence:**

Guifang Ouyang

fyyouyangguifang@nbu.edu.cn

Rui Mao

[mr3749@163.com](mailto:mr3749@163.com)

^+^ These authors contributed equally to this work

# Supplementary Figures and Tables

## Supplementary Table

###### Supplementary Table 1 Four pairs of CA-CDA primers were designed by DNAMAN

| **Target** | **Method** | **Primer** | **Sequence (5´→3´)** |
| --- | --- | --- | --- |
| ITS2 | CDA | CA-MF-1 | TCGATGATTCACGTCTCTTGGTTCTCGCATC |
|  |  | CA-MR-1 | ATCTTTGAACGCACGAAACGACGCTCAAACAG |
|  |  | CA-MF-2 | ATTCGATGATTCTCTCTTGGTTCTCGCATC |
|  |  | CA-MR-2 | CTTTGAACGCACGAAACGACGCTCAAACAG |
|  |  | CA-MF-3 | TTCAAAGATTCGTCTCTTGGTTCTCGCATC |
|  |  | CA-MR-3 | CGCACATTGCGCGAAACGACGCTCAAACAG |
|  |  | CA-MF-4 | CGCAATGTGCGTTCTCTTGGTTCTCGCATC |
|  |  | CA-MR-4 | CCCTCTGGTATTGAAACGACGCTCAAACAG |

## Supplementary Figure

**
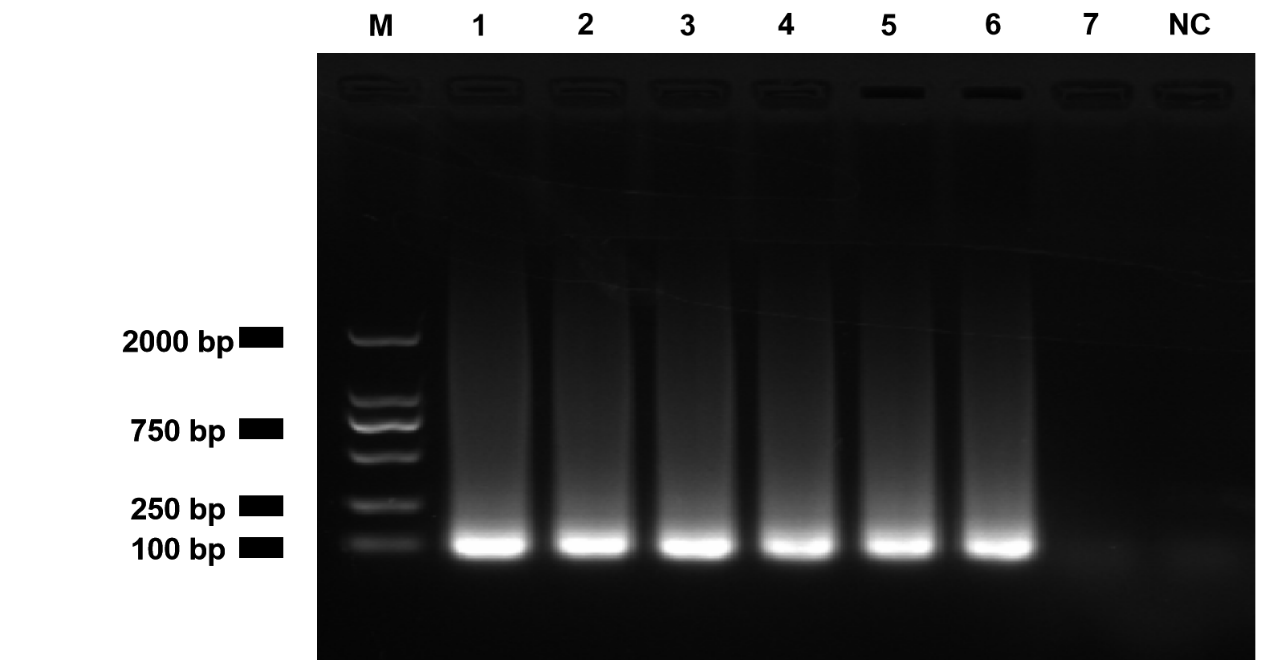
**

**Supplementary Figure 1.** Agarose gel electrophoresis of products by CA-OL-CDA amplification. M: DL 2000 DNA Marker;1-7: 6.2×10-1 ng/μL, 6.2×10-2 ng/μL, 6.2×10-3 ng/μL, 6.2×10-4 ng/μL, 6.2×10-5 ng/μL, 6.2×10-6 ng/μL, and 6.2×10-7 ng/μL of Candida albicans DNA; NC: negative control.
